# It is high time we changed our habits. Chest pain: when ECG is not enough and echo makes the difference

**DOI:** 10.1186/2036-7902-7-S1-A6

**Published:** 2015-03-09

**Authors:** M Frameglia, E Frongia, P Riolfi, B Genco, C Bertrand, A Zocca, C Menini, L Adami, C Bellunato, G Arcoleo,G Battizocco

**Affiliations:** Emergency Department Orlandi Hospital, Bussolengo, Verona Italy

**Keywords:** Chest Pain, Pneumothorax, Holistic Strategy, Apical Window, Lung Point

## Background

In the diagnostic flow chart of chest pain the normality of 12 lead ECG is considered the crucial point.

## Objective

The combining ECG with point of care ultrasound allows to establish the correct diagnosis better and faster.

## Patients and methods

Case History. A 31 years old man was in his office when he felt a sudden chest pain, localized in the left hemi-thorax and radiated to the back. In a few minutes an ambulance reached the place. The vital signs were good. A 12 lead ECG was registered and sent to the hub hospital. The answer was: “ECG normal. No signs of acute coronary syndrome. Transport the patient to the nearest local hospital”. Meanwhile the patient started feeling better. At the arrival to the emergency department the triagist assigned to the patient a green code (“will eventually need help but can wait for others”) and wrote: “ Non specific chest pain. Anxiety.” After two and half hours the poor guy called the doctor and told him that the pain was going much better and he wanted go home. The doctor took the patient in a room, switched on the echo machine and spent 2 minutes to make the right diagnosis. The heart was normal but was visualized only from the subcostal window. Para-sternal and apical windows were impossible to find. The reason was quite simple. A left pneumothorax was present, as documented by the absence of normal lung sliding and lung point sign. (see video clips 1-5) . A chest tube was then inserted and the problem solved.

## Results

The clinical strategy was wrong. It is not enough to perform a 12 lead ECG and send it to a remote cardiologist to be sure that everything is right On the other side a point of care echography examination proved to be so powerful that in a very short time the right diagnosis was made [1, 2]. It is not enough to exclude a STEMI by a 12 lead ECG.

## Conclusion

It’s time we changed our habits! Reality is complex and the modern problem- based approach , which means a more holistic strategy, including point of care echography, saves time, reduces mistakes and improves outcomes. Instead of rigid protocols used by operators that act like computers, we need competent people able to turn on first their brains and possibly their echo machines.

## Informed consent

The study was conducted in accordance with the ethical standards dictated by applicable law. Informed consent was obtained from each owner to enrolment in the study and to the inclusion in this article of information that could potentially lead to their identification.
